# Effect of Neonatal Intensive Care Unit (NICU) Humidity on Neonates: A Systematic Review

**DOI:** 10.7759/cureus.58524

**Published:** 2024-04-18

**Authors:** Ashok Kumar Urakura, Ravi Gajula, Greeshma Reddy Kankanala, Rakesh Kotha, Suresh Babu Mendu, Neelam Harsha

**Affiliations:** 1 Pediatrics, Government Medical College Vikarabad, Vikarabad, IND; 2 Pediatrics, Government Medical College Siddipet, Siddipet, IND; 3 Neonatology, Osmania Medical College, Hyderabad, IND; 4 Neonatology, Niloufer Hospital, Hyderabad, IND

**Keywords:** skin, neonatal development, preterm, low birth weight, humidity, neonates

## Abstract

This study explores the role of room humidity levels in the neonate intensive care unit (NICU) and how they impact their growth and development in this fragile stage. The study considers seven relevant studies that have explored this factor in different settings. Humidity's role in developing neonatal conditions, such as respiratory distress and fungal infections, is also elaborated on. For the literature review, the study utilized PubMed, Embase, and Scopus databases to guarantee comprehensive findings on the role of NICU room humidity on neonates. By examining these studies' evidence, the research highlights the paramount need to ensure that the room has adequate moisture, as exposure to less desirable humidity levels increases mortality and the severity of morbidity rates among neonates. The fact that the majority of NICUs lack humidity control, which is required, stimulated us to conduct this review.

## Introduction and background

Neonatal care stands at the intersection of advanced medical technology and compassionate, meticulous attention to the unique needs of premature and critically ill infants. At the 18th week of gestation, the fetus's skin becomes keratinized. At 32 to 34 weeks of gestation, the stratum corneum, responsible for regulating evaporative heat loss and transepidermal water loss (TEWL), has reached its complete maturity. Babies who are born before 30 weeks of gestation have skin that is not fully developed, which makes them more susceptible to cold and dehydration. Because of TEWL, infants delivered at 26 weeks or earlier will experience high fluid loss from their skin (about 150 milliliters per kilogram per day). This will decrease urine output and electrolyte imbalances and increase fluid maintenance. Water loss through evaporation expends calories and generates more heat than the infant can produce at rest.

Because they are exposed to aerated environments, the skin of preterm neonates grows quite quickly after birth. By two to three weeks after birth, regardless of the length of gestation, the skin of a newborn is functioning in a manner comparable to that of a term baby. The World Health Organization (WHO) estimates that about 15 million babies are born prematurely every year [1]. The complications of prematurity are the main reasons for death among young children below five years old worldwide.

The environmental conditions for neonatal units are critical, and as such, the heated and humid air gauged by these fragile patients' health and wellness is maintained to optimal levels. Humidity regulation is a cornerstone of neonatal care, given the unique vulnerabilities of premature infants. The protective skin barrier of preterm neonates is underdeveloped, causing dehydration, irritation, and breakdown [2]. The cells are made up of different protein coatings. When the intact humid coat is missing from these fragile epidermal layers, the risk of them splitting off, resulting in wound injury, increases. In addition, the development of dermatitis and infections is heightened [3]. There is also the significant consideration of medium water loss, which occurs in the skin's outermost layer in low-birth-weight neonates, often resulting in substantial morbidity [4].

Beyond their impact on skin integrity, humidity levels profoundly influence respiratory health in neonates. Poor humidity can harmfully alter the delicate balance that the body can maintain for the respiratory tract surface mucosal interface and may impair or inhibit ciliary action, so the airways are inadequately protected against pathogens and particles [5]. Apart from this, the breathing patterns of preterm babies are disrupted, and their immature organs become predisposed to various infections, such as respiratory distress syndrome (RDS) [6]. RDS is usually associated with premature birth and involves problems with surfactant production and compliance of the lungs [7]. In addition, substandard humidity conditions worsen respiratory tract infections, which widely affect premature infants [8].

Humidity management remains one of the most critical factors in neonatal care. Still, despite the growing community awareness of its contribution, there needs to be a uniform understanding of the most appropriate humidity levels for various gestational ages and clinical conditions [9].

## Review

Methods

**Figure 1:**
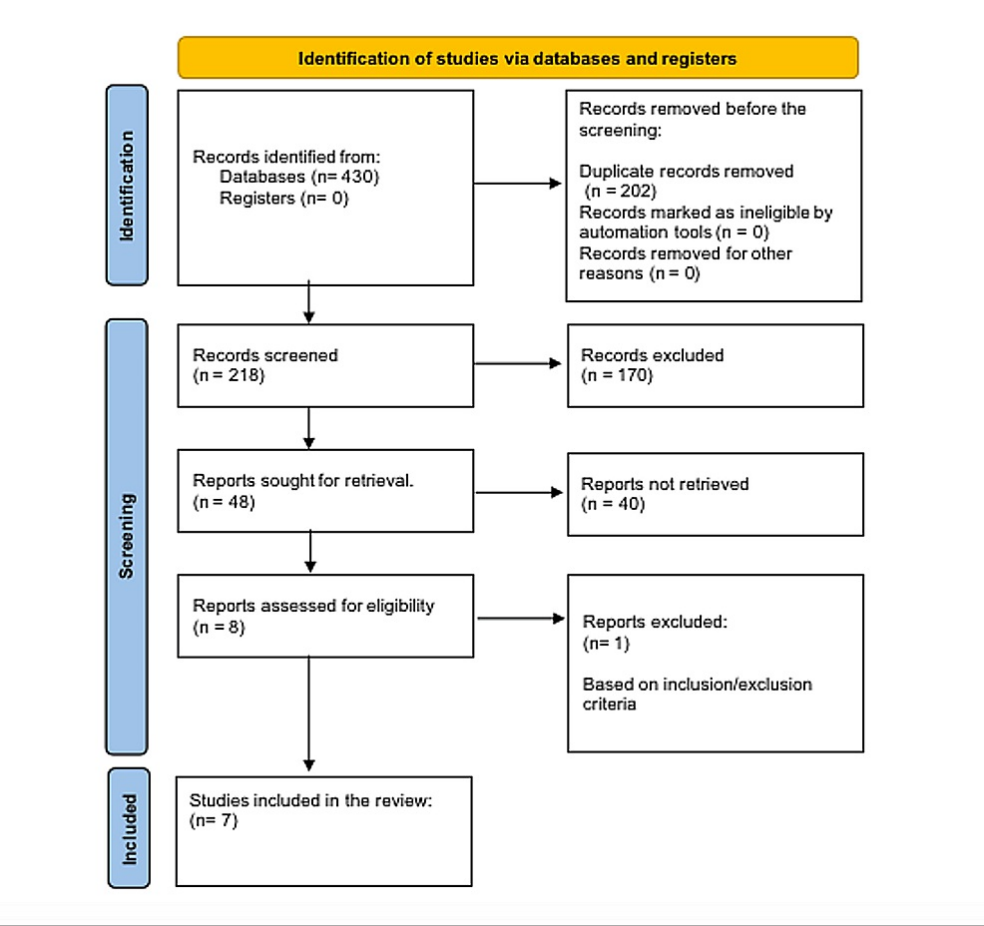
PRISMA diagram for the identification of studies PRISMA: Preferred Reporting Items for Systematic Reviews and Meta-Analyses

A comprehensive search strategy (Table 1) of electronic databases, such as PubMed, Embase, and Scopus, returned an initial group of 430 articles published in the January 2000-March 2024 range. Using stringent criteria, the pool of results was restricted to seven similar matching studies that met the predetermined inclusion criteria (Table 2). This research examined the consequences of humidity levels on neonatal well-being issues, e.g., respiratory distress syndrome and bronchopulmonary dysplasia. These findings were published in the English language. The screening process included two reviewers' separate and independent reviews of the titles, abstracts, and full texts to achieve consensus on conflicts and conclude that the representative titles should be included in the review. Information extraction involves examining numerous components, including basic information, the number of patients involved, and the measures of the effects. The Preferred Reporting Items for Systematic Reviews and Meta-Analyses (PRISMA) flow diagram tool was utilized to document the various phases of the literature search procedure (Figure 1). We registered our systematic review under PROSPERO with ID 532984.

**Table 1:** Database search strategy

Search	Strategy
#1 Search	“Neonatal intensive care unit” AND (humidity OR “moisture control”) {MeSH terms}
#2 Keyword search	Neonatal intensive care unit" AND (humidity OR "moisture control" OR "air quality" OR "environmental health") {MeSH terms}
#3 Keyword search	Preterm infants" AND ("humidity management" OR "humidity control" OR "humidity levels" OR "humidity monitoring")
#4 Keyword search 3	“NICU temperature” OR “temperature regulation” OR “thermal management” {Title/ Abstract}
#5 Keyword search 4	Premature AND neonates AND (outcomes OR “health outcomes”) AND humidity {Alternative terms}
#6 Keyword search 5	NICU AND (“environmental factors” OR “facility design”) {Truncation}
#7 Keyword search 6	“Neonatal infection” AND (“outbreak” OR “nosocomial infection”) AND NICU {Truncation}

**Table 2:** Sampling criteria

Inclusion criteria	Exclusion criteria
Original research studies examining the impact of humidity levels on neonatal health outcomes.	Case reports, case series, and case studies
Studies focusing on neonates in neonatal intensive care units (NICUs) or similar settings	Studies conducted on animals or in vitro models
Studies published in English.	Studies not published in English
Studies report outcomes related to respiratory distress syndrome (RDS), bronchopulmonary dysplasia (BPD), skin integrity, or other relevant neonatal health outcomes.	Studies published before January 2000
Studies were conducted between January 2000 and March 2024.	

Quality Assessment of the Studies

The quality assessment of the seven studies in this research involves various methods tailored to the study design. In this case, which evaluated observational studies like the neonatal intensive care unit (NICU) room humidity investigation, the Newcastle-Ottawa scale (NOS) was selected to determine the methodological strength and level of risk of bias. This analysis tool examines the characteristics of the research project, including study design, comparability, and outcome assessment, offering a systematic approach to ranking study quality. This ensures a comprehensive overview of the quality of a study. In addition, the study reported randomized clinical trial findings using the Risk of Bias 2 (ROB 2) tool to investigate the methodological quality and risk of bias in the study design, implementation, and analysis. The distinctive feature of this evaluation is the intensive study, which verifies the credibility and accuracy of the reported observations in each of the studies. As per the respected scale, our included studies were of high quality on that particular scale. As with the case for systematic reviews, the reviewing process was carried out cautiously, including only high-quality and relevant studies. This made the overall synthesis more robust. The systematic review was of high quality as per AMSTAR-2 (A Measurement Tool to Assess Systematic Reviews). GRADE (Grading of Recommendations, Assessment, Development, and Evaluations) is a transparent framework used for case series that is of high quality.

Description of Studies

Samartharam et al. (2021): This interventional study explored the impact of maintaining a humidity of 70-80% and a temperature of 32°C in a room-based incubator on premature neonates' outcomes. The experiment took the entire room, converted it into an incubator, and found out that the babies were born either with a low birth weight (LBW) or were in good condition. It suggested that closed nursing care may be considered a more beneficial practice. This study will help in maintaining humidity through drip water curtains. It is most suitable for developing countries [10].

Vashishtha et al. (2012): This study described a fatal outbreak of *Trichosporon asahii* sepsis in a NICU, highlighting the importance of environmental factors, such as humidity control in preventing such outbreaks. This shows how humidity control is one of the factors that need to have their focus shifted during the prevention of such outbreaks. The research emphasized the significance of managing humidity (i.e., low humidity) for a safe NICU [11].

Horns (2002): This comparative study explored the thermal stability of extremely-low-birth-weight (ELBW) infants placed into two varying microenvironments: the standard radiant warmer with modified hood as intervention and RINCE, which is an experimental reduced-intervention nursing caregiving environment. It was revealed that the RINCE considerably impacted environmental and ambient temperature variability, relative humidity variability, and infant temperature stability. This impact was significant, especially during and after the everyday caregivers' activities in the NICU [12].

Thomas et al. (2010): This study aimed to make a seasonal map of temperature conditions in the air, radiant and evaporative types, and moisture levels of ambient air throughout the year, analyzing a NICU nursery. Seasons substantially impact the variation in the relative humidity levels; therefore, it is essential to consider humidity alongside ambient air temperature while leading even a NICU [13].

Liu et al. (2023): This study investigated the association between climatic conditions and term birth weight by applying the data recorded at the Republic of Cyprus birth register. The conclusion was that in the case of a mother expecting a child during either early or late pregnancy, increased ambient temperature exposure leads to lower body weight, indicating the influence of climate change on perinatal results [14].

Kao et al. (2022): This systematic review considered the predictive power of interventions for coexisting diseases in LBW infants in humidity-enriched medium environments. This review also provided recommendations for adopting high-humidity conditions and postulated physiological indicators and outcomes in related investigations in neonatal care [15].

Meyer et al. (2001): This clinical trial compared radiant warmer and incubator care for preterm infants in light of temperature control and weight gain. After looking at abdominal temperatures and identifying outcomes, the study observed differences between the groups, suggesting the advantages of the first use of radiant warmers [16].

Table 3 summarizes the summary of the included studies.

**Table 3:** Summary of results

Study	Type of study	Year	Country	Objective	Comparative group	Conclusion/outcome
Samartharam et al. [10]	Interventional study	2021	India	To evaluate the role of humidity in managing premature neonates in a rural setting	Existing literature	Open nursing care with a room temperature of 32°C and humidity of 70% resulted in excellent outcomes for premature neonates.
Vashishtha et al. [11]	Case study	2012	India	To describe an outbreak of *Trichosporon asahii* sepsis in a neonatal intensive care unit	None	*Trichosporon asahii* was identified as the causative organism, sensitive to amphotericin-B but resistant to fluconazole; the outbreak was linked to a laminar flow unit.
Horns et al. [12]	Randomized controlled study	2002	USA	To compare two microenvironments and nursing caregiving on thermal stability of meager-birth-weight (ELBW) infants	Control bed vs. RINCE	The RINCE significantly improved ambient temperature variability, RH variability, and the infant's peripheral and delta temperature stability.
Thomas et al. [13]	Observational study	2010	USA	To create a thermal map of ambient air, radiant and evaporative temperatures, and humidity throughout the NICU nursery by season	None	Seasonal thermal differences were observed, particularly in humidity and evaporative temperatures; room temperature alone does not reflect the total thermal environment.
Liu et al. [14]	Observational study	2023	Cyprus	Identifying critical exposure windows to examine the association between ambient climatic factors and term birth weight	None	Higher ambient temperature exposure during early and late pregnancy is associated with lower birthweight and susceptibility in populations during sensitive exposure windows.
Kao et al. [15]	Systematic review	2022	China	To explore the predictive effects of interventions for comorbidities performed on very-low-birth-weight preterm infants in high-humidity environments	None	Recommendations for high humidity intervention for preterm infants <30 weeks of gestation or <1,000 grams birth weight to improve physiological indicators and avoid complications
Meyer et al. [16]	Randomized controlled study	2001	New Zealand	To compare radiant warmer and incubator care for preterm infants concerning temperature control and weight gain	Radiant warmer vs. incubator	Initial use of radiant warmer after birth showed benefits; differences in abdominal temperatures on day one and outcomes, with cautious interpretation due to the sample size.

Discussion

Various studies have offered valuable insights into the crucial role of room humidity within the NICU and its profound impact on neonatal outcomes. Samartharam et al. found that a humidity level of 70-80% was maintained at 32°C for premature babies. Researchers found that these had established a scenario comprising a balance of humidity and temperature in which neonates did well [10]. Through this, we may adequately manage room humidity, which will work with the temperature control process and can be designed to provide enhanced neonatal care. Moreover, Vashishtha et al.'s (2012) study predicted the detrimental effects of the lack of humidity control in NICU intensive care units. In their report, they observed an outbreak of Trichosporon asahii sepsis among newborns that was believed to have been caused by some factors, such as improper humidity levels in the environment [11]. This points to the fact that proper dehumidification is vital in NICUs to overcome the risks of diseases acquired in hospitals and provide safety for susceptible neonates. Problems with regulated humidity levels can create an environment that promotes the growth of pathogens, posing considerable danger to neonates and increasing the development of harmful outcomes.

In addition, Horns (2002) contrasted two microenvironments - one within and the other outside a unit - and assessed the effect level on two LBW infants. The study corroborated the fact that a nursing caregiving environment that had patients' temperature regulated and required minimal intervention significantly improved ambient temperature variability and stability, hence underscoring the necessity of using optimal humidity, which goes along with the process of promoting thermal stability and reducing the risk of encountering complications in vulnerable neonates [12]. This proves the importance of humidity control as a crucial component in the NICU's neonatal care policies and protocols. 

Furthermore, Thomas et al. (2010)'s study provides crucial clues into the seasonal humidity fluctuations experienced within the NICU environment [13]. Research by Liu et al. (2023) contributed to our understanding of the association between ambient temperature exposure and birth weight during specific pregnancy periods. The findings of this study revealed that when a pregnant female is exposed to high ambient temperatures during the critical period window, the likelihood of giving birth to a baby with a lower birth weight is higher [14]. This case links developing fetuses' fragility to environmental factors like humidity and temperature. This is even during their sensitive stages of growth and development. That is why keeping the humidity levels optimal becomes an assigned task in preventing adverse postnatal outcomes arising from the baby's exposure to environmental conditions. Therefore, a sound practice of moisture control can attenuate the possible adverse effect of environmental factors on fetal development. It may consequently create better neonatal health outcomes.

In light of these findings, the importance of humidity management in the NICU cannot be overstated. The article by Kao et al. (2022) was also relevant since it functioned as a systematic review of the existing literature that strongly suggested the beneficial effects of high-humidity environments for long-term health outcomes for preterm infants. According to the data findings, the correlation between humidity control and optimal physiological data was explored, and any ailments in neonatal care could be eliminated [15]. This scientific research exposed the importance of humidity management, as evidenced by their recommendations to fulfill the humidity level requirements of preterm infants, which is one aspect worth considering in neonatal care protocols. 

Moreover, this was confirmed by the data presented by Meyer et al. (2001) when the research was carried out to define the ability to survive in a heated or ventilated environment [16]. Although both groups received additional humidity, the group with the radiant heater test demonstrated signs of efficient temperature adjustment and fewer reports of adverse effects [16]. Temperature-controlling systems have become necessary to balance recycling processes, which strive to minimize the environmental impact. Improving neonatal care and positive neonatal outcomes in the NICU depend on this and may positively influence the child's development if proper care and attention are provided. Healthcare facilities possess a moral obligation to provide functioning atmospheric conditions, particularly in the NICU, to guarantee excellent or superior medical services to preterm infants of LBW.

Comparison to Other Studies

Different from the previous research conducted to identify the relevance of room humidity in NICUs, the discoveries of Samartharam et al. (2021), which point to the necessity of controlling temperature altogether with humidity, are in harmony with already existing views [10,17]. By so doing, this is what other studies [12] tried to emphasize since their research stated that to attain thermal balance and hence improve the mortality rate, the issue of variability should be strongly controlled [12]. However, Vashistha et al. (2012) disclosed this case of *Trichosporon asahii* sepsis in a newborn infant [11]. Despite the unfortunate incident, a high relative humidity level in the NICU could have prevented this. Interestingly, these listed values emphasize the necessity of tight humidity control in NICUs, as reducing the chances of getting these infections is a significant step toward assuring the overall health of preterm infants [18].

In addition, the platforms upon which we can build this knowledge are the research conducted by Thomas et al. (2010) and Liu et al. (2023) [13,14]. They emphasize the necessity of establishing humidity levels, which function based on seasonal changes and their impact on the primary health of infants [19]. Appreciating this diversity implies conducting extensive research and brainstorming on the most relevant programs to handle this issue throughout the year [20]. On the contrary, a systematic review by Kao et al. (2022) focused on the potential advantages of the early stage, starting with enhancing staff productivity. Despite many efforts and therapies, humidity still occupies a dominant factor in the environment given to premature babies and has substantial significance [15].

In addition, according to a study by Meyer et al. (2001), the need for moisture is examined in both types of settings, with radiant warmers and incubators. Although radiator warmers were added to the setting, the radiator-warmed group demonstrated an advantage in terms of temperature management, improved comfort levels, and better-monitored indicators [16]. This stresses the necessity for healthcare institutions to increase their focus on moisture and temperature control so that better NICU regimens can be made and the potential of all neonates can be normalized [21]. 

Limitations of the Systemic Review

One limitation of this systematic review is the potential for inadequate literature on room humidity and its role in neonatal care. Many researchers emphasize the importance of non-genetic factors in neonatal health outcomes, temperature stability, or air quality [22]. On the other hand, this literature, specifically the part related to room humidity, could be narrow and insufficient. The need for well-designed studies investigating the relationship between room humidity and the health outcomes of neonates could be a barrier to the depth and scope of the systematic review's results. This limitation may arise due to several reasons:

Research focus: Previous studies carried out in neonatal care could only have addressed environmental factors, e.g., thermal regulation or infection prevention measures [23]. From there, a few studies will research room humidity as a factor in neonatal health outcomes.

Publication bias: Studies where the impact of room humidity on neonatal health is reported to be harmful or nil studies may most likely have a problem with publishing, and hence, there is a probability for bias to be inclined towards studies demonstrating significant effects. This may become a bias factor, narrowing the scope of the literature source for the systematic review.

Research priorities: The direction and depth of funding in neonatology may subtly affect the research matters being examined and published studies. If room humidity has yet to be included in research to determine if it should be considered a priority study, there is a joint systematic review that may be less to consider.

Study design limitations: The existing studies that link room humidity and infant health outcomes may differ in many aspects, including study design, sample size, and methodological principles [24]. The mixed nature of the studies will likely impact the summing up and arriving at effective results due to the systematic review since the methodology may need to be unified.

Geographic variation: Research on the connection between humidity in a room and neonatal outcomes may differ across the world's regions. This may be because of the variations in the quality of healthcare facilities, environmental conditions, and the availability of resources [25]. This geographic heterogeneity could lead to contradictions in the outcome and constraints on the possibility of broadening the sample.

## Conclusions

This systematic review provides valuable insights into the role of room humidity in neonatal care and its implications for neonatal health outcomes. Although limited acknowledgment of neonatal care or intervention and humidity is found throughout the literature, it nevertheless reveals the critical role of moisture in improving the health outcome for preterm or LBW infants. We have found that averted neonatal respiratory distress syndrome can be alleviated by reducing fluctuations in humidity. Nonetheless, the need for more research on the above topic obliged further studies on its impact on a comprehensive scale. Besides the other environmental variables, clinical professionals should be more proactive in monitoring and maintaining optimal humidity levels to create safer neonatal care surroundings. By implementing evidence-based thinking, clinicians can elevate the level of care in neonatal units and thus enhance overall health outcomes for neonates. Further research would be vital to uncover the possible meaning and develop practice protocols that will be created based on the evidence to improve the surrounding conditions and prevent neonatal disorders. Lastly, even though it is challenging, room humidity needs to be changed based on country, season, and gestation.
